# Improving access to health commodities by strengthening the supply chain Management workforce: the case of Namibia

**DOI:** 10.1186/2052-3211-7-S1-O6

**Published:** 2014-12-17

**Authors:** Erin Hasselberg, Lazarus Indongo, Tonata Ngulu, Kennedy Kambyambya, Benjamin Ongeri

**Affiliations:** 1 Partnership for Supply Chain Management, JSI Research & Training Institute/John Snow, Inc. Boston, MA, USA; 2 Division of Pharmaceutical Services, Ministry of Health and Social Services, Windhoek, Namibia; 3 Central Medical Stores, Ministry of Health and Social Services, Windhoek, Namibia; 4 National Medicine Policy Coordination, Ministry of Health and Social Services, Windhoek, Namibia; 5 Management Sciences for Health, Supply Chain Management System (SCMS), Windhoek, Namibia

## Background

The Namibia Ministry of Health and Social Services (MoHSS) approached People that Deliver (PtD) for support in addressing supply chain management (SCM) workforce challenges at its central and regional medical stores (CMS/RMS). PtD leveraged the expertise of member organizations CapacityPlus and SCMS to provide technical assistance in planning, deployment, training and retention of the SCM workforce; document the process and lessons learned; and draft a case study on the process for PtD to share globally.

## Method

The MoHSS and PtD partners collaborated on four key interventions to address SCM workforce challenges. These included developing a SCM competency framework, identifying the number and types of supply chain personnel required using the Workload Indicators of Staffing Need (WISN) tool, conducting targeted capacity building starting at the central medical store through the Supply Chain Performance Improvement (SCPI) program, and identifying context-specific incentives to encourage staff retention using the Discrete Choice Experiment (DCE) activity.

## Results

Preliminary results indicate that there are opportunities and political will to reduce role overlap between pharmacists, pharmacist assistants, and clerks at CMS/RMS and to tailor in-service and pre-service training programs based on the newly drafted competency frameworks for these cadres. By the time of the conference additional results from the November 2013-September 2014 collaboration will be presented on the number and types of staff needed to fulfil these three cadres at CMS/RMS, the package of salaries and incentives most likely to attract and retain them in these positions, and progress against a set of key performance indicators.

## Discussion

This collaboration focused on three cadres within the CMS/RMS level of the supply chain; however, in the future expanding the application of activities to hospital and clinic levels will produce a more thorough picture of the SC workforce. The tools utilized in the Namibia pilot will be shared in order to apply this unique approach in other countries; currently Mozambique, Burkina Faso, and Liberia have plans to introduce a similar collaboration. The collaboration was possible due to the coordinating power of the PtD Initiative and is suggestive that similar opportunities for future innovative pilots in strengthening the SC workforce exist.

## Lessons learned

This collaboration was successful due to a combination of MoHSS support and leadership, and the leverage of PtD in convening global expertise in HR and SCM. USAID regarded these activities as a “smart investment” given the minimal additional funding required and strategic use of in-country partner presence and tools.

